# Food Environment Typology: Advancing an Expanded Definition, Framework, and Methodological Approach for Improved Characterization of Wild, Cultivated, and Built Food Environments toward Sustainable Diets

**DOI:** 10.3390/foods9040532

**Published:** 2020-04-22

**Authors:** Shauna M. Downs, Selena Ahmed, Jessica Fanzo, Anna Herforth

**Affiliations:** 1 Department of Urban-Global Public Health, School of Public Health, Rutgers University, Newark, NJ 07102, USA; sd1081@sph.rutgers.edu; 2 Sustainable Food Systems Program, Department of Health and Human Development, Montana State University, Bozeman, MT 59717, USA; 3 Berman Institute of Bioethics, Nitze School of Advanced International Studies and Bloomberg School of Public Health, Johns Hopkins University, Washington, DC 21205, USA; jfanzo1@jhu.edu; 4 Department of Global Health and Population, Harvard T.H. Chan School of Public Health, Harvard, University Boston, MA 02125, USA; aherforth@hsph.harvard.edu

**Keywords:** sustainable diets, natural food environments, built food environments, socio-ecological framework, climate change

## Abstract

The food environment is a critical place in the food system to implement interventions to support sustainable diets and address the global syndemic of obesity, undernutrition, and climate change, because it contains the total scope of options within which consumers make decisions about which foods to acquire and consume. In this paper, we build on existing definitions of the food environment, and provide an expanded definition that includes the parameter of sustainability properties of foods and beverages, in order to integrate linkages between food environments and sustainable diets. We further provide a graphical representation of the food environment using a socio-ecological framework. Next, we provide a typology with descriptions of the different types of food environments that consumers have access to in low-, middle-, and high-income countries including wild, cultivated, and built food environments. We characterize the availability, affordability, convenience, promotion and quality (previously termed desirability), and sustainability properties of food and beverages for each food environment type. Lastly, we identify a methodological approach with potential objective and subjective tools and metrics for measuring the different properties of various types of food environments. The definition, framework, typology, and methodological toolbox presented here are intended to facilitate scholars and practitioners to identify entry points in the food environment for implementing and evaluating interventions that support sustainable diets for enhancing human and planetary health.

## 1. Introduction

Nourishing a growing population in ways that support human and planetary health is one of the greatest challenges of the Anthropocene. Malnutrition in all its forms, including overweight, obesity, undernutrition, and their coexistence, is the leading cause of death globally and affects every country [1,2,3]. Most countries (88%) are experiencing a coexistence of multiple burdens of malnutrition [1,2,3], much of which is associated with diets high in saturated fat, sugar, highly processed foods, and meat, while being low in fiber, fruits, and vegetables [4]. At the same time, human activities are having unprecedented impacts on the Earth and its systems, including through greenhouse gas emissions linked to climate change [5,6]. It is anticipated that climate variability and human-induced climate change will continue to exacerbate malnutrition [3], food insecurity [7], and hunger [8], leading to an even greater burden of disease attributed to diets. 

Recently, the three pandemics of obesity, undernutrition, and climate change have been described as a global syndemic given their clustering in time and place, interactions at biological, psychological, and/or social levels, as well as common large-scale drivers and determinants [3]. 

While food production, processing, distribution, and preparation, and our food choices place stress on the environment, these food system processes are critically dependent on ecosystems and their services [9,10]. Food production practices are threatening the very resource base which they are dependent on including the availability of quality soils, water resources, and biodiversity [11]. Several planetary boundaries of environmental thresholds recognized as safe operating spaces for humanity have already been crossed [5] with serious effects on human and planetary health [12]. The stability of the resource base which our food security is dependent on is threatened by climate change [13]. There is a clear need to improve the resilience of food systems to climate shocks as well as other external pressures of global change [9]. Concurrently, industrialization, development, and globalization of food systems have enhanced the connectivity and interdependence of food supply chains resulting in emerging issues of food quality, food safety, problems in transparency, and concentrating resources in fewer hands [14]. The increase in complexity of food supply chains is further associated with food and agricultural sectors to be governed separately with broken linkages between production, consumption, distribution, processing, and waste management [14].

The concept of sustainable diets, or healthy diets from food systems that support planetary health, has gained momentum in recognition of the interconnected challenges in the way we produce, procure, prepare, consume, and waste food [15,16]. There is a recognized need to shift dietary patterns in ways that protects the environment while feeding a growing population with healthy, culturally appropriate, acceptable, and desirable food [17,18,19,20,21,22,23,24]. Sustainable diets take into consideration that food choices are part of complex and dynamic food systems that exist at multiple scales from the local to the regional, national, and global, with the goal of supporting both environmental and human wellbeing [14,22,25,26]. Typically, sustainable diets are characterized on the basis of four key dimensions including ecological, economic, human health, and sociocultural and political dimensions [23,27,28,29]. There are increasing efforts for promoting sustainable diets with a focus on interventions that influence consumer behaviors (see Box 1 for types of interventions to promote sustainable diets [30,31]).

Box 1 **Types of interventions to promote sustainable diets.**

Interventions to promote sustainable diets can target different entry points in the food system from production, processing, and distribution, to the food environment, food preparation, consumption, and waste [18]. In addition, interventions to promote sustainable diets can be classified along a continuum of intensification on the basis of level of influence. On the low-influence side of the continuum are efforts for informing and empowering to those that seek to guide and influence [30]. On the high-influence side are efforts for incentivizing, discouraging, or restricting [30]. In addition to levels of intensification, interventions that promote sustainable diets can be characterized on the basis of the stakeholder group implementing the changes including policy makers and the private sector [30]. For example, policy interventions that involve incentives or disincentives to support sustainable diets may involve taxation of food with unsustainable attributes [32,33]. Food environment interventions for informing and empowering consumers to make food choices aligned with sustainable diets may involve product labeling with environmental information such as food production practices that support mitigating greenhouse gas emissions [34]. Interventions within the food environment aimed at guiding and influencing consumers to make healthy and sustainable food choices may involve positive positioning of food with healthy and sustainable attributes in retail settings, reduced plate sizes in buffet-style restaurants and school cafeterias, and signage about reducing food waste in eateries [35]. Apart from the food environment, interventions for informing and empowering at the individual-choice level may involve food and nutrition education to motivate behavioral change for making more sustainable food choices such as increasing dietary diversity, eating more locally-sourced plant-based and nutrient-dense food, and reducing consumption of ultra-processed foods [36].

In this paper, we assert that the food environment is a critical place in the food system to implement interventions to support sustainable diets and address the global syndemic of obesity, undernutrition, and climate change, because the food environment contains the total scope of options within which consumers make decisions about which foods to acquire and consume. However, the current literature is missing several key aspects that prevent the comprehensive characterization of food environments in order to develop effective interventions to support sustainable diets. First, current food environment frameworks do not explicitly integrate sustainability or links to sustainable diets. Second, there is a need for a comprehensive description of the different types of food environments that consumers have access to, including wild, cultivated, and built food environments, in order to more effectively identify entry points to modify food environments to support sustainable diets [37]. Last, there is a need to identify and validate metrics, tools, and methodologies for measuring the various parameters of each type of food environment [38]. In recognition of these needs, the overall goal of this paper is to present the following objectives: (1) expanded definition of the food environment that provides clarity to previous definitions as well as integrates the attribute of sustainability; (2) a framework positioning the food environment within the food system based on a socio-ecological model; (3) a food environment typology that includes both natural (wild and cultivated) and built (informal and formal market) food environments; and (4) a methodological approach accompanied with potential tools and methods for measuring the elements of the food environment (availability, affordability, convenience, promotion and quality, and sustainability properties of food and beverages) based on the food environment typology in low-, middle-, and high-income countries in the context of global change. 

## 2. Methodological Approach

The objectives of this concept paper were addressed on the basis of the following: (1) multiple literature reviews; (2) extensive field observations carrying out food environment research in diverse socio-ecological contexts in low-, middle-, and high-income countries by the study team; (3) interactions with field experts during workshops, symposium, and conferences on the topic of food environments; and (4) classroom experiences teaching graduate students on the topic of food environments. Collectively, our study team has carried out food environment research in a range of low-, middle-, and high-income countries including rural and tribal communities in Asia (China [39,40,41,42,43], India [44,45,46], Nepal [28], and Myanmar [47]), Africa (Kenya [48], Senegal [49], and Tanzania [50]), and North America (Waskaganish, Quebec [51] and Flathead Reservation, Montana [22,36,52,53,54,55,56]). Our field work in these communities have included mixed-methods to capture the key elements of the food environment types and how consumers interact with those environments. We have further had semi-structured interactions about food environment definitions, frameworks, and methodologies during workshops and conferences including: (1) Building a Food Environment Community of Practice Workshop (November 2016, Honolulu, HI, USA); (2) Food Environment Metrics to Support Dietary Needs in the World Food Center Workshop: Aligning the Food System to Meet Dietary Needs: Fruits and Vegetables (June 2017, Davis, CA, USA), (3) the Society for Ethnobiology conference symposium on ‘Indigenous Peoples and Food Systems in Transition: How can ideas from ethnobiology inform work on food environments’ (May 2019, Vancouver, BC, Canada), and (4) the CGIAR Agriculture for Nutrition and Health (A4NH) Consultative Food Environment Workshop (November 2019, Addis Ababa, Ethiopia). Our classroom experiences teaching graduate students on the topic of food environments, such as the ‘State of the Food Environment: Policy, Measurement, and Practice’ course, have informed our thinking for this concept paper by highlighting the state of the field, gaps, and common areas of uncertainty with regards to key food environment concepts that we seek to clarify in this paper. Below we outline the specific approaches applicable to each key objective of this concept paper. 

For Objective 1, we arrived at an expanded definition of the food environment based on our previous definition through a literature review on food environment definitions [57,58,59,60,61] coupled with our experiences carrying out food environment research and the goal to provide clarity regarding misconceptions of what the food environment is and how the food environment can support sustainability. For Objective 2, we developed the socio-ecological framework of the food environment within the food system through a literature review on socio-ecological models [57,62,63,64,65] and interactions of food environments with diets, communities, cultures, markets, politics, governance, and ecosystems [66,67,68,69,70,71]. For Objective 3, we developed the food environment typology that includes both natural and built food environments through observations of diverse food environments at our study sites coupled with a literature review on food procurement [37,59,72,73,74]. For Objective 4, we arrived at the multi-faceted methodological approach of measuring food environments with potential tools through a literature review on existing methods used for measuring built food environments [57,75,76,77,78] along with potential tools we have used during field research measuring wild and cultivated food environments that draw from the fields of anthropology and ethnobiology [22,47,53]. 

## 3. An Expanded Definition and Framework of the Food Environment That Integrates Sustainability

The term food environment first emerged in ecology to refer to the context and attributes of food chains of various species, such as the nutritional quality and abundance of the food supply of different herbivores in ecosystems [79]. At the turn of the 21st century, the concept of the food environment expanded and began to also refer to the built environment in human societies [66] with a focus on characteristics of neighborhoods [67,68,69] and practices within institutional settings, particularly schools [70] and workplaces [71] in high-income countries. The concept of the food environment increasingly began to be applied to examine linkages between the built food environment, diets, and chronic disease in high-income countries as the nutrition and public health communities embraced a socio-ecological model of examining the multi-faceted and interactive factors that influence food choices [62,63], particularly of health disparate populations [64]. Socio-ecological models were initially developed in the 1920s with the application of systems thinking from ecology to examine human behavior and advanced as conceptual models for evaluating human health and development in the 1970s [65]. 

As the concept of the food environment further broadened to be applied in low- and middle-income countries (LMICs), in addition to high-income settings, we identified the need to provide a definition of the food environment and its properties for diverse settings [57]. We thus defined the food environment as: the consumer interface with the food system that encompasses the availability, affordability, convenience, and desirability of foods [57]. We further presented the need of the food environment definition, framework, and methods to be applicable in wild and cultivated food environments that characterize where households directly procure food in many LMICs [37]. 

**Figure 1:**
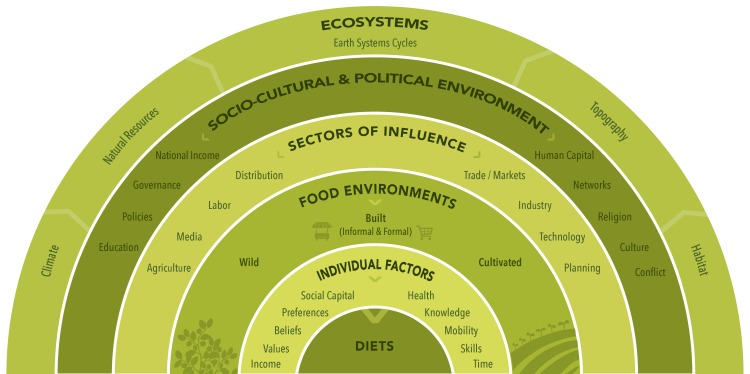
Positioning the food environment within the broader food system based on a socio-ecological model. The layers closest to diets (i.e., individual factors and food environments) include the structures and processes which individuals directly interact with in their immediate surroundings. The outer layers (i.e., sectors of influence, socio-cultural and political environment and ecosystems) are the more distal drivers influencing food environments, individual factors and diets.

**Figure 2:**
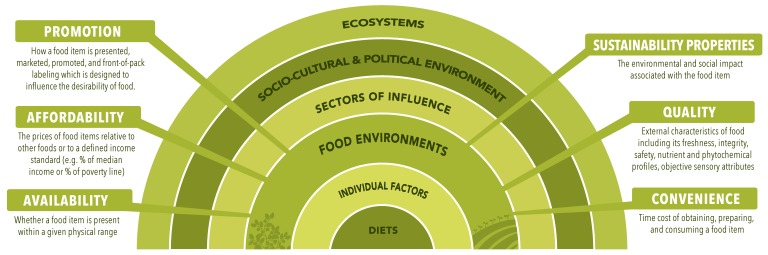
Descriptions of the food environment key elements. The key elements of the food environment within the food system include the availability, affordability, convenience, promotion and quality, and sustainability of foods and beverages in wild, cultivated, and built spaces.

In this paper, we build on previous definitions of the food environment [57,59,80] to include the property of sustainability as well as emphasize different types of food environments accompanied by a graphical representation based on a socio-ecological model (Figure 1) alongside descriptions around previously identified properties of availability, affordability, and convenience of foods (Figure 2). The revised and expanded definition of the food environment that we propose, that is intended to be applicable in low-, middle-, and high-income countries, is: the consumer interface with the food system that encompasses the availability, affordability, convenience, promotion and quality, and sustainability of foods and beverages in wild, cultivated, and built spaces that are influenced by the socio-cultural and political environment and ecosystems within which they are embedded. 

Figure 1 is a graphical representation of the expanded definition of the food environment that we propose based on a socio-ecological model. In this graphical representation, the layers closest to diets (individual factors and food environments) contain the structures and processes that individuals directly interact with in their immediate surroundings, also known as the micro- and meso-systems [65]. Beyond the food environment, there are sectors of influence impacting the availability, affordability, convenience, promotion and quality, and sustainability properties of foods and beverages, which individuals do not generally interact with. These sectors of influence include trade, markets, industry, technology, planning, distribution, labor, agriculture, and media. Beyond these sectors of influence are the broader socio-cultural and political environment, or macro-system, that include factors such as governance, national income, culture, conflict, religion, policy, education, networks, and human capital. Ultimately these various scales of systems are embedded within the ecosystem, which is regulated by earth system cycles and climate, and is dependent on natural resources, habitat, and topography. 

Aligned with the socio-ecological model of food and beverage intake, our expanded definition of the food environment includes only external factors that describe the total array of foods and beverages in the environment [57,62]. Other definitions have included individual-level factors influencing food choice such as taste and preferences [59,81], which we consider to be factors that interact with the food environment rather than being part of the food environment per se. In addition, various other definitions have included broad policy, cultural, and socio-economic drivers as part of the food environment [58,61]. We recognize the usefulness of assessing these drivers within efforts to improve food environments at policy level, but focus our definition on describing and measuring the actual foods and beverages in the food environment. 

Our previous definition of the food environment included desirability as an element to describe the external factors that influence the desirability of food including advertising, product placement, and food quality [57]. Due to frequent misinterpretation, and the use by other authors of the term “desirability” to include individual-level factors such as preferences [59], we revise the term “desirability” as “promotion and quality” in order to provide clarity on this food environment element.

Secondly, we add sustainability properties as an element of the food environment. In current dialogue about sustainable diets, consumers are encouraged to make dietary choices that are more sustainable, i.e., with regard to the environmental, economic, socio-cultural, and human health impacts of food production, preparation, transport, retail, storage, processing, and waste [18,23]. While sustainability is addressed through changes throughout the food system [58], the food environment is ultimately where consumers interface with the food system and make choices toward sustainable diets. As the incentives and disincentives such as prices and promotions within the food environment influence food choices, which can subsequently impact environmental outcomes (e.g., greenhouse gases (GHGs), water footprint, loss of biodiversity, etc.), there is an urgent need to examine sustainability properties of foods and beverages within the food environment research space.

Sustainability is part of each product’s properties, and to the extent that it can be observed by consumers, it can influence food choices. For example, in a wild food environment, people may choose not to consume a certain animal as bush meat if it is endangered due to its sustainability properties. Or, if the only tomatoes available in a given formal market food environment are shipped from thousands of miles away and packaged in plastic, those sustainability properties can interact with individual-level values to determine choices. In this sense, sustainability is similar to quality, such that both are external characteristics that describe a particular food item, and both may have tradeoffs with price or convenience. 

The sustainability properties of foods and beverages can be characterized on the basis of the four key dimensions of sustainability including ecological, economic, human health, and sociocultural and political dimensions [27,28,29,36]. Previously, we identified 32 sub-dimensions of sustainability attributes of various components of food systems including sustainability properties of foods that support sustainable diets as well as sustainability attributes of food environments [23]. Table 1 provides an overview of the different sustainability properties of foods and beverages that support sustainable diets. However, it is important to note that there are often tradeoffs among different sustainability properties. For example, locally produced foods are often viewed as being more sustainable given the perceived shorter transportation distances from farm to market leading to lower greenhouse gas (GHG) emissions [82]. However, the majority of GHG emissions stem from the production step of the supply chain. Thus, the way foods are produced likely influence sustainability more than where they were produced. At the same time, many of the producers participating in local food systems adopt sustainable and diversified farming practices such as moderating or abstaining from the use of fertilizer and other agrichemical input, cover cropping and intercropping, implementing crop rotations, and cultivating diverse crops and crop varieties [82]. 

Unlike the other properties of the food environment (availability, affordability, convenience, and promotion and quality) that are readily perceived by the consumer, sustainability properties are often not transparent to the consumer. Visible signals or labels regarding sustainability attributes of foods are limited or absent in most food environments. Organic labels are one example of how foods can be visibly identified according to some sustainability criteria (the organic movement is motivated by consumer demand for food that is produced more sustainably, and by consumer demand for perceived food safety (both with regard to pesticide use)); the use of plastic or other packaging is another. However, typically limited information is provided about how any particular food or beverage item was produced. Furthermore, even with perfect information, different aspects of environmental and social impacts often make it difficult for consumers to rank and decide which products are more or less sustainable. In formal markets, there are often tradeoffs between various aspects of sustainability, such as organic bananas wrapped in plastic, vs. conventional bananas that are free of packaging. In this sense, sustainability properties are similar to nutritional qualities, such that they encompass many aspects, which individual consumers may value more or less, and are important to make visible through the use of labels and information. Despite the limited ability to describe sustainability properties of most foods at present, we include it as an aspect of the food environment because it is a product property distinct from the others (price, convenience, and promotion and quality). We advocate for improved product information so that consumers can make choices based on the sustainability properties of each food and beverage option in their food environments. However, we acknowledge that not all consumers demand information related to sustainability properties and it may only appeal to a small segment of the population. Moreover, there is a possibility that some consumers may be overloaded with this additional information. Ultimately, explicitly integrating sustainability as a parameter of the food environment should enable the design and implementation of interventions that enhance diets and nutrition outcomes while mitigating climate risk [3] in ways that support both human and planetary health.

## 4. Food Environment Typology

Typology refers to the study of different concepts/items through classifying phenomena based on commonalities or differences. The goal of creating and examining typologies is to better understand the conditions and factors of a given phenomenon and how they relate to each other [83]. Much of the food environment research to date has focused on describing the formal built food environment in high-income countries and its association with health outcomes [74]. More recently there has been an increase in the food environment research and literature in LMICs, yet these studies have still largely focused on the built environment [72]. This may be due in part to the differences between disciplines that are most often involved in the characterization of built food environments (e.g., nutritionists, public health researchers, etc.) compared to those that conduct research examining the natural food environment (e.g., ecologists, ethnobiologists, agronomists, etc.). Although the built environment is the most common food environment type in high-income countries, where the majority of the food environment research has been conducted, the same is not true of many rural and low-income settings where a notable proportion of food is acquired through foraging and agriculture [59]. Approximately 475 million farms globally are small (<2 hectares of land), with the majority (~92%) situated in LMICs [84,85,86]. It is estimated that these smallholder farms produce approximately one third of the food supply [87,88]. Although most smallholder households are net food buyers, they also rely on their own production to meet their energy and nutrient needs. Food environment assessments and interventions must therefore also take into account natural food environments in order to have relevance in rural settings for many populations living in LMICs [37].

We built upon the existing food environment and food system literature [37,58,59] to identify the different types of food environments that communities interact with globally, including in LMICs. More specifically, we used the existing literature, as well as our previous work examining food environments in diverse socio-ecological settings, to describe the different types of food environments in which consumers can access food. Our goal here is solely to describe the various types of food environments that exist globally as a foundation for future research, such as characterizing these different types of food environments and how changes in them can affect diets. The creation and application of a food environment typology allows for a more comprehensive understanding of the socio-ecological determinants of diets as well as enabling comparative analysis between places and across time. 

**Figure 3:**
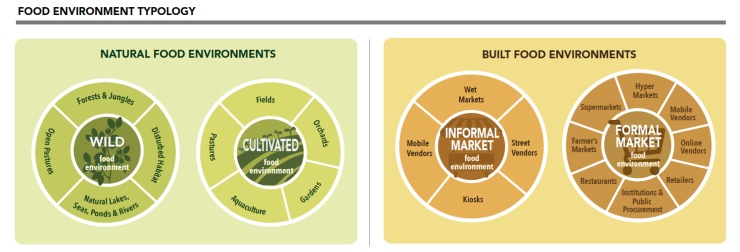
Food environment typology. There are two overarching types of food environments comprising the food environment typology including natural and built environments. These further comprise of wild, cultivated, informal market, and formal market food environments.

We identify two overarching types of food environments comprising the food environment typology: natural and built environments (Figure 3). Natural food environments, also known as subsistence food environments [37], include wild and cultivated food environments. Wild food environments include forests and jungles, disturbed habitat, open pastures, and aquatic areas. Table 2 describes the food environment types and associated sub-types in additional detail, including the food outlets or access points that are considered within each. While we use the term “wild”, we recognize humans have an extensive history of influencing landscapes even before the origins of agriculture [89] and that wild to cultivated food environments vary along a continuum of management and intensification [90]. In contrast to wild food environments, cultivated food environments have greater management. Cultivated food environments include fields, orchards, closed pastures, gardens, and aquaculture from which consumers directly procure food. Other agricultural systems such as large commercial farms are not typically part of the cultivated food environment as consumers do not directly procure food from these systems. Rather, food from large commercial farms makes its way through supply chains prior to being sold to consumers in the built food environment. Given that food environments are where people access food for their own consumption, the cultivated food environment refers to food production for own-household consumption; it does not refer to food cultivation for sale.

Built food environments, also known as market or retail food environments [37], include informal and formal markets. Food supply chains link the production, processing, and distribution of food to these food environments. Informal market food environments are those that are often not regulated through formal governance structures [91] and include wet markets, street vendors, kiosks, and mobile vendors. Traditional and modern-to-traditional food supply chains feed into informal built food environments [92]. Formal market environments are those that are regulated through formal governance structures where sellers can publicly advertise their locations and prices and includes hypermarkets, supermarkets, and retailers as well as farmer’s markets and restaurants. Modern and traditional-to-modern food supply chains provide the foods that consumers are able to access within the built food environment [92]. 

**Figure 4:**
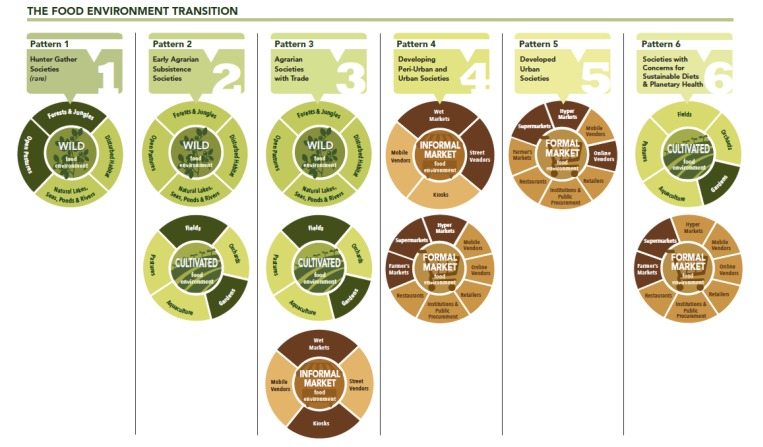
Transition of food environment typology with development. The types of food environments that communities and countries have access to may shift over time with development. This figure depicts how the food environment types change aligned to Popkin’s nutrition transition [4]. A sixth pattern of food environment types was added to indicate a transition to societies with concerns for sustainable diets and planetary health (Pattern 6).

Individuals, households, and communities may have access to various types of food environments at a given time point and this may shift with time. For example, the types of food environments can vary temporally, such as based on season as well as over time with global change [93,94,95,96]. Box 2 highlights how the types of food environments a community within a country has access to and relies on may further vary with development over time, in what we term the food environment transition (Figure 4).

Box 2 **Shifts in the types of food environments people have access to with development.**

The five societal patterns of food procurement highlighted in Popkin’s [97] nutrition transition framework are associated with a transition of the types of food environments that communities have access to (Figure 4). Specifically, the food environment transition aligned to Popkin’s nutrition transition depicts a shift from hunter gatherer lifestyles (Pattern 1) to agriculture societies that are found in low- and middle-income countries (Patterns 2 and 3), to upper–middle income countries that are characterized as peri-urban and urban developing societies, to high-income developed urban societies (Pattern 5). We added a sixth pattern to the change of food environment types with a food environment transition indicate societies with concerns for sustainable diets and planetary health (Pattern 6).

Pattern 1 includes societies that interface predominantly with wild food environments. Such communities exist in many parts of the world yet are experiencing rapid change [95]. The domestication of plants and animals gave rise to agrarian societies that rely on food from wild and cultivated food environments (Pattern 2); such agrarian societies are presently found in low and low–middle socio-demographic index (SDI) countries in parts of Asia, Africa, and Latin America. They can also be found in countries that experience disruptions to trade due to conflict, disease outbreaks, and extreme events. As agrarian societies grew and began to specialize, they gave rise to a surplus of food that allowed for trade (Pattern 3). With trade, these agrarian societies began to rely on informal food markets in addition to natural food environments. This societal pattern resembles middle SDI countries in many parts of rural Africa, Asia, and Latin America today, where consumers acquire food primarily through wild, cultivated, and informal food markets [39,98,99,100,101]. The 19th and 20th centuries saw agricultural intensification globally with mechanization, selective breeding, and increased agricultural production that allowed for greater societal specialization away from the farm (Pattern 4). In this societal pattern, consumers rely on informal and formal food environments including wet markets, street vendors, hypermarkets grocery stores, and convenience stores. This pattern characterizes many high–middle SDI countries at present. With increased trade, development, urbanization, and technological advances over the past 60 years, coupled with changes in consumer preferences, high SDI developed urban societies are primarily reliant on formal market food environments characterized by supermarkets, hypermarkets, discounters, and a rapid increase in internet retailing (Pattern 5). While each of the food environment transition patterns can support aspects of sustainable diets, increased awareness and changes in societal values for sustainability are leading to a societal pattern where consumers are increasingly seeking healthy and sustainably-sourced foods (Pattern 6). Pattern 6, which we describe as societies with concerns for sustainable diets and planetary health, revitalizes integration of earlier food environment typologies, including urban agriculture. Although there are few, if any, examples of this pattern on a national scale, there are examples of cities and regions in high SDI countries that are shifting towards this pattern such as Tuscany and the Apulia region in Italy [102] and Western Australia [103]. These changes in types of food environment with shifts in development provide a general description of a phenomenon; communities and countries do not have to transition in a linear fashion through each pattern as they undergo socioeconomic development. It is also possible that within a given country or community, multiple types and patterns of food environments exist.

Table 3 provides an overview of the key food environment elements (availability, affordability, convenience, promotion and quality, and sustainability properties of food and beverages) within each type of food environment. Future research is called for to build the evidence to support these descriptions in diverse socio-ecological contexts and modify them as appropriate.

## 5. Methodological Approach and Potential Tools for Measuring Food Environment Properties Based on Typology

To date, the food environment literature has largely focused on geographical analysis as the main mode of measurement including geospatial data of food outlets as well as checklists, interviews and questionnaires, economic appraisal, market baskets, and inventories [57,59,72,76,77]. These methodological approaches have been critiqued for being too narrow in scope and for inadequately measuring the way in which people are truly exposed to, and interact with, their food environments [104]. While many of the methodological approaches used in high-income countries have the potential to be applied to LMIC contexts, they have several limitations. In LMICs, consumers interact with wild, cultivated, and informal built food environments in addition to the formal built environments that often typify the food environments in high-income countries. Although in recent years there has been an increase in the food environment literature in LMICs, a common understanding of best practices for measuring food environments does not yet exist. This is a critical gap in the literature which limits the ability to design, implement, and evaluate the effect of food environments and food environment interventions on dietary quality, nutrition, and health outcomes. The lack of best practices and standardized protocols for measuring food environments that are applicable in LMICS limits the ability to compare food environments across contexts and through time in response to interventions, policies, and global change. 

Here, we provide a methodological approach for measuring food environments that are applicable in diverse contexts accompanied by a review of potential existing methods and tools. Given that consumers based in many countries generally interface with multiple types of food environments, and that food environments are multi-faceted, multiple methods are needed for their comprehensive measurement. First, measurement of the food environment should include the key elements of availability, affordability, convenience, promotion and quality, and sustainability. Secondly, given the existence of different types of food environments including wild, cultivated, informal built, and formal built food environments, measurements should be inclusive and appropriate for each type. Within a given type of food environment, the most appropriate methods to measure food availability, affordability, convenience, promotion and quality, and sustainability properties may differ based on location/outlet. For example, a tool for measuring availability in the built food environment may not be appropriate for wild or cultivated food environments. Rather, methodological approaches drawing from ecology and disciplines that focus on human interactions with the natural world including ethnobotany, ethnoecology, and agroecology would be more suitable for measuring elements of wild and cultivated food environments [37]. Third, measurements of the food environment should include both objective as well as subjective or perceived measures [57,59,80]. Objective measures remove bias and variability in evaluating food environments, whereas subjective measures take into account the experience and reality for consumers. Fields such as anthropology, ethnobotany, and ethnoecology have a long history of characterizing perceptions of the surroundings and can be drawn on to create subjective measures to accompany objective measures. For example, Appendix A provides a tool for measurement of the sustainability properties of foods in the food environment that can be scored by trained raters, whereas Appendix B provides a parallel tool on perceived sustainability properties of foods in the food environment based on the experience of individuals interacting with a specific food environment. 

In Table 4, we draw from the food environment and sustainable diets literature [27,36,59,72,76,77,105,106,107] as well as ecology, ethnobotany, environmental sciences, and other fields to provide an overview of potential methods for measuring the food environment. This overview includes objective and perceived availability, affordability, convenience, promotion and quality, and sustainability properties of foods and beverages in wild, cultivated, informal, and formal food environments [36]. Table 5 provides an overview of specific tools that have been used to measure the different elements of food environments. It is important to note that the methodological overview we provide serves as an initial exploration of methods for measuring objective and perceived elements of different food environment types given that there are currently several gaps in the existing methods where new tools or methods need to be developed. By measuring both objective and subjective or perceived elements, it is possible to characterize the external food environment as well as the way in which individual-level factors (e.g., sometimes referred to as the personal food environment) influence how a given individual interacts with the food environment. 

## 6. Discussion 

This paper seeks to advance food environment research and practice through presentation of the following: (1) an expanded definition of the food environment with accompanying descriptions of the elements of availability, affordability, convenience, promotion and quality, and sustainability properties of foods, (2) a food environment framework based on a socio-ecological model, (3) overview of a food environment typology with a description of the elements of availability, affordability, convenience, promotion and quality, and sustainability properties of foods within each type of food environment and, (4) a methodological approach with potential methods for measuring different elements of the food environment based on type. By more comprehensively characterizing food environments in different countries and regions, and measuring them using appropriate methods, interventions and policies aimed at improving food choices that are aligned with sustainable diets can be better tailored to the contexts in which people live. By improving the food environments that consumers interact with, and linking these to sustainability, there is potential to improve dietary patterns towards supporting both human and planetary health. 

One of the advantages of characterizing the different types of food environments is that it can help to describe how food environments shift over time. In LMICs, globalization of food trade, including foreign direct investment, has led to shifts in the availability and type of food outlets and types of food and beverages [174,175,176,177]. Food supply chains in LMICs have undergone rapid transformations in recent years [92,178], leading to marked changes in the foods that consumers have access to within their food environments [179]. Characterizing how the types of primary food environments shift over time is particularly important in LMICs where consumers often source foods from various different food environments. Another advantage to characterizing food environment types is that it can help to describe the changes among food environments across seasons, including in the context of climate variability and change. Climate variability and change can affect which foods are produced, the quantity and quality of those foods, as well as their availability, affordability, and accessibility [180,181]. 

To date, there has been limited integration of sustainability into the food environment research space. The sustainability properties of foods and beverages are an important parameter of the food environment, particularly within the context of sustainable diets and planetary health, yet little information is available to consumers. Information on sustainability properties needs to be clearer to enable consumers to make food choices toward sustainable diets and to allow researchers to measure this parameter of the food environment. Moreover, additional research is needed to elucidate the sustainability properties of many commonly consumed foods specific to the context where they were produced and consumed [182]. Without this information, the incorporation of sustainability properties into food environment assessments will be limited. 

### Limitations

Although we used existing literature and extensive field experience to inform the development of the food environment typology described in this paper, and the attributes of foods within each of its types, limited literature on these different food environment types means that this work is largely conceptual. Future research should aim to develop new, and refine existing, methodological approaches for characterizing different food environment types. Those methods can be subsequently used to provide empirical data to inform the refinement of our food environment typology. 

Although we include sustainability properties of foods as a parameter of food environments, due to the fact that consumers only interact with sustainability via making choices within the set of foods they can access in their food environments, further work needs to be conducted to identify key methods to assess sustainability properties of foods. 

## 7. Conclusions

This paper provides a food environment typology that includes both natural and built food environments as well as an updated food environment definition relevant to LMIC and sustainable diets. We provide an overview of the food environment properties in each of the food environment types, and a toolbox of objectives and subjective tools and metrics to measure them. This work provides the foundation for future empirical research to comprehensively measure various types of food environments across different settings with the view to informing the development of interventions and policies aimed at encouraging the consumption of healthy and sustainable diets.

## Figures and Tables

**Table 1:** Sustainability attributes of foods and beverages that support sustainable diets (Adapted from Ahmed et al. (2019) [36]).

Dimension of Sustainable Diets	Sustainability Attribute of Foods and Beverages
**Ecological Dimension**	**Production quality:** The food supports production systems that cultivate for nutritional quality (crop quality). **Biodiversity, agrobiodiversity, and ecosystem services:** The food supports conservation and maintenance of biodiversity and agrobiodiversity as well as associated ecosystem services. **Sustainable agriculture:** The food supports sustainable agricultural practices and sustainable intensification that limit pesticide, herbicide and fertilizer use. **Local and seasonal foods:** The food supports the procurement of foods that are in season and are local. **Clean energy:** The food supports the use of clean energy and green or sustainable technologies. **Soil, land, and water conservation and protection:** The food supports the procurement of food in ways that prevent contamination of soil, land, and water resources such as protecting watersheds from pollutants. **Low GHGE and climate resilience:** The food supports production methods with relatively low GHGEs; designing and managing for agricultural systems for climate change/climate resilience.
**Economic Dimension**	**Distribution, supply chains, and transport:** The food supports direct sales between producers and consumers. **Food loss and waste:** The production and preparation of the food minimizes loss of food waste across the food system from farm through fork. **Food packaging:** The food has minimum food packaging and/or encourages recycling. **Food system livelihoods:** The production of the food promote livelihoods to support stakeholders in the food system from on farm and throughout food value chains. **Farmers’ markets and local food systems:** The production of the food recognizes the importance of local food systems including farmers’ markets, community supported agriculture (CSA), food cooperatives, and food hubs. **Food storage and preparation:** The production and preparation of the food avoid resource-intensive food storage of cold chain items and high-energy preparation such as the use of a microwave.
**Human Health Dimension**	**Food safety:** The food is safe and prevents foodborne illness, contamination, negative health influence of agriculture and diseases linked to chemicals and pesticide use. **Plant-based and nutrient-dense foods:** The food is plant-based and nutrient dense foods such as fruits, vegetables, and legumes. **Macro- and micro-nutrient adequacy:** The food contributes essential macro- and micro-nutrients to the diet.
**Socio-Cultural and Political Dimension**	**Equity issues:** The production of the food supports equity in the food system including on-farm, in market, trade, distribution, food service, and policy sectors. **Labor:** The food supports safe labor conditions and standards for workers in the food system. **Animal welfare:** The food supports healthy, comfortable, well nourished, and safe conditions for animals raised for livestock.

GHGE: Greenhouse gas emissions.

**Table 2:** Food environment typology describing food environment types. A description of the specific types of food outlets and access points within wild, cultivated, informal, and formal food environments.

Food Environment Type	Food Environment Sub-Type	Description of Food Environment Sub-Typology
**Natural food environments**		
Wild food environments	Forests and jungles	Forests, jungles, woodlands, marshlands, and other intact natural habitats in which people can procure food.
	Disturbed habitat	Roadsides, vacant lots, and other areas where weeds and other feral plants grow.
	Open pastures	Land areas including prairies and savannahs in which wild and domesticated animals roam and graze.
	Natural lakes, seas, ponds, and rivers	Oceans, lakes, and rivers from which people procure food.
Cultivated food environments	Fields	Small-, medium-, and large-scale farm areas in which farmers cultivate crops for own consumption.
	Orchards	Fruit, nut, etc. trees or shrubs planted for food production.
	Pastures	Farming areas for livestock in which domesticated animals roam and graze.
	Gardens	Home, kitchen, community, and rooftop gardens cultivated for food.
	Aquaculture	Breeding, rearing, and harvesting of fish, shellfish, and plants (e.g., seaweed).
**Built food environments**		
Informal market food environments	Wet markets	Daily or weekly markets that sell primarily fresh foods often directly by the producers and in open air settings.
	Street vendors	Unlicensed vendors that are positioned on streets and sidewalks who sell a variety of foods.
	Kiosks	Kiosks are informal boutiques or small stalls/shops that sell food.
	Mobile vendors	Vendors that travel (e.g., by motorcycle, truck, etc.) to a given location (e.g., rural village) to sell food. These vendors are only present at specific times of the day, week, or month and do not have permanent infrastructure in the location.
Formal market food environments	Supermarkets	Supermarkets, grocery stores, small-scale independent grocers, co-ops, and specialty stores.
	Hypermarkets	Supercenter, megastore, big box stores, or other large retail stores that sells both food and non-food goods and is most often part of a chain of stores.
	Retailers	Mom and pop shops, corner stores, bodegas, etc. that sell food.
	Farmer’s markets	Formal markets that often occur periodically that sell foods directly from farm to consumer.
	Restaurants	Casual dining, upscale dining, fast food, and cafes where prepared meals are sold for sit-down service, take-out, or delivery.
	Institutions and Public procurement	Cafeterias and food vending machines in schools, workplaces, childcare facilities, hospitals, and recreation centers.
	Mobile vendors	Formal street vendors such as food trucks that have a license to operate.
	Online vendors	Online vendors that sell and deliver groceries and prepared foods (e.g., Uber eats), to one’s home.

**Table 3:** Conceptual description of the key elements of the food environment (availability, affordability, convenience, promotion and quality, and sustainability) based on the type of food environment.

Food Environment Element	Food Environment Type			
	Wild	Cultivated	Informal Built	Formal Built
**Availability/Diversity**				
Wild plants and animals represent local biodiversity	x			
Diversity of plants and animals is dependent on region (e.g., agro-climatic zone; socio-ecological conditions)	x	x		
Seasonally available F&V	x	x	x	
Limited diversity in smaller food outlets			x	
Branded and unbranded processed food, and sometime ultra-processed foods			x	
Variation across seasons			x	
May have a vast diversity of food available in all seasons from different locations				x
Availability of foods may differ based on neighborhood SES				x
Availability of minimally processed and ultra-processed foods				x
**Affordability**				
No monetary exchanges	x	x		
Trading of goods	x	x		
Staples relatively inexpensive			x	x
Nutrient-rich foods (e.g., F&V and ASF) relatively expensive and/or price is highly seasonally variable			x	
Processed foods packaged in small packages to increase affordability			x	
Many ultra-processed snack foods, ready meals, and fast foods made with cheap ingredients are inexpensive				x
Fruits and vegetables, seafood expensive				x
Pay high premiums for specialty/niche foods and locally produced or organic foods				x
**Convenience**				
Can be labor and time intensive to hunt or gather	x			
In some situations can be highly convenient (e.g., when wild fruits are in season)	x			
Labor and time intensive during growing season		x		
Processing of staples and food preparation time sensitive		x		
Independent (non-chain) fast food and street vendors offer convenience foods such as ready-to-eat snacks and meals			x	
Distance to markets can be long and road access limited in rural areas			x	
Numerous chained fast food outlets, casual dining, and other restaurants				x
Improved infrastructure with cars and public transport increase market access				x
Processing of ingredients along with ready-to-eat and ready-to-heat foods reduces cooking time				x
Increased use of online delivery				x
**Promotion & Quality**				
Marketing of food non-existent	x	x		
Promotion of food limited to farmer-targeted programs or extension services		x		
Food is fresh by definition when wild harvested	x	x		
Crop quality is variable		x		
Branding and advertisements in print in newspapers and posters			x	
Signs in stores, markets, and buildings			x	
Verbal promotion on radios			x	
Variable freshness/quality and high food losses are common due to lack of cold chains and unstable storage conditions			x	
High level of food promotion through television, print, web, billboards, and sports sponsorships				x
High amount of labeling, nutrition facts panels, health claims, ingredients in stores, and on menus				x
Food safety standards generally ensure safe food				x
Quality of perishable food is typically high due to intact cold chains, but can be variable (e.g., convenience stores vs. supermarkets)				x
**Sustainability Properties**				
Support of ecosystem services (soil, land, and water protection)	x			
Low carbon footprint	x			
Sustainability dependent on abundance of supply in ways that do not deplete integrity of resource base (e.g., through overharvesting)	x			
Food consumed are local and seasonal	x	x	x	
Carbon and water footprint dependent on production practices		x		
Soil health dependent on production practices		x		
Food loss high in LMIC contexts		x		
Land tenure issues		x		
Relatively low levels of packaging			x	
Food system livelihood and equity issues			x	
Food safety, quality, and regulatory issues			x	
High levels of food loss due to inadequate storage conditions			x	
High amounts of packaging				x
High levels of food waste				x
Food system livelihood and equity issues				x
Food miles can be high				x
High carbon and water footprint of some foods (e.g., beef)				x
Biodiversity may be restricted and pesticide use high due to focus on marketability				x
Foods sourced from different locations				x
High-energy food storage of cold chain items				x
Less transparency regarding food production practices				x

ASF: Animal source food; F&V: Fruits and vegetables; LMIC: Low- and middle-income countries; SES: Socio-economic status.

**Table 4:** Objective and perceived methods for measuring food environment properties by typology.

Food Environment Measurements/Methods	Food Environment Type			
	Wild	Cultivated	Informal Built	Formal Built
Description of types of foods sold at each food outlet [108,109]				
Diversity inventories [36]				
Inventories of foods sold by food outlet type and associated metrics [75,76,78]				
Number, location, density, and proximity of food outlets in defined geographical areas [76,110]				
Direct observation of food outlet location, type, and density [78,111,112]				
Assessing commercial or government business listings of registered food businesses [78]				
Ratio of fresh to processed food or healthy to unhealthy foods [75,78]				
Ratio of shelf space allocated to specific types of foods (fruits and vegetables, ultra-processed foods etc.) within stores [78,108,113]				
Seasonal calendars of food availability [114,115]				
Transect and plot inventories with associated diversity metrics [116,117]				
Free listing of foods [118]				
Participatory social mapping of food environment [119]				
Perceptions of food availability [118]				
Photo elicitation [120]				
Cost of diet analysis [50,121,122]				
Cost of food basket [76,123]				
Expenses involved in agricultural production [124]				
Market surveys to assess food prices [75,78]				
Perceptions of food cost and affordability [118]				
Accelerometers to measure time and energy spent foraging and preparing foods [125]				
Accelerometers/pedometers/GIS mapping to assess distance to food acquisition (GIS, travel time, etc.) [76,125]				
Direct observations of time spent acquiring and preparing foods [126]				
Perceived time spent acquiring or preparing foods [126,127,128]				
Time use surveys to examine time spent foraging or preparing foods [126,127,128]				
Analysis of toxins, bacteria, etc., and adulteration of foods [129]				
Direct observations of marketing/social marketing (e.g., radio announcements, billboards, etc.) [75,76]				
Direct observations of labelling [75]				
Food safety ratings of food outlets [130]				
Nutrient/phytochemical analysis of foods (direct analysis or using food composition tables) [76,131]				
Promotion and education material near to food products [75]				
Physical measurements of shelf space and prominence of specific foods [78]				
Recall of exposure to marketing/social marketing [132]				
Sensory surveys [53]				
Analysis of contaminants or residues present in food sold				
Assessment of acquisition of local or seasonal foods				
Direct observations of labels such as “organic”, “local”, “integrated pest management”, “free range”, “fair trade”, product origin, etc. [133]				
Direct observations of use of packaging				
Life cycle assessment of foods [96]				
Measurement of food losses and waste [134]				
Surveys to assess farm management practices [135]				
Sustainable dimensions food environment rating framework [23]				
Interviews/surveys to assess awareness of product origin, procurement of local or seasonal foods [28,136,137]				

Color Coding: 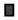 Objective measure 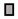 Perceived measure; BOGO: Buy one get one; CH4: Methane; CO_2_: Carbon dioxide; FoodAPS: USDA’s National Household Food Acquisition and Purchase Survey; GHGe: Greenhouse gas emission; GIS: Geographic information system; HEISB: Healthy eating indicator shopping basket; IPM: Integrated pest management; N_2_O: Nitrous oxide; NEMS: Nutritional environment measures survey.

**Table 5:** Overview of Specific Tools to Assess Different Food Environment Elements.

Tools	Availability	Affordability	Convenience	Promotion and Quality	Sustainable Properties	References
Nutritional Environment Measurement Survey (NEMS) (versions: restaurants, stores, corner stores, vending, grab and go, and Rudd Center Revised version) ^†^						[138,139,140]
Nutritional Environment Measurement Survey-Perceived (NEMS-P)						[138,141]
Short Form Audit Instrument for Assessing Corner Store Healthfulness						[142]
INFORMAS food retail						[107]
Healthy Eating Indicator Shopping Basket (HEISB)						[143]
Freedman Food Store Survey						[144]
Baltimore Healthy Stores Project Store Evaluation Form						[145,146]
Food Environment Availability and Cost Measures						[147]
ProColor Diversity Tool						[36]
Community Health Environment Scan Survey (CHESS)						[148]
Measurement of healthfulness of food retail stores						[109]
Food Environment Classification Tool						[78,112]
Retail Food Environment Index (RFEI)						[78,149]
Food Availability and Marketing Survey						[150]
Community Food Security Assessment Tool						[151]
Nutrition Environment Assessment Tool						[152]
New Jersey Child Health Study Survey						[153]
Teens food service data collection instrument						[154]
Survey of healthy activity and eating practices and environments in Head Start (SHAPES)						[155]
Food and beverage Marketing Assessment Tool for Settings (FoodMATS)						[156]
Restaurant Menu Checklist						[157]
Perceived Availability of Healthy Food Questions						[158]
Neighborhood Food Assessment Tool						[159]
Health Empowerment Zone Grocery Store Checklist *						[160]
Grocery Store Audit Tool and Fast Food Restaurant Audit Tool						[161]
Shannon diversity Modified Functional Attribute Diversity						[116,117]
Cost of Diet						[121]
Cost of Nutrient Adequacy						[50]
Cost of a Recommended Diet						[122]
Nutritious Food Price Index						[122]
INFORMAS food price module						[105]
Cost of a healthy and sustainable food basket						[162]
Price Comparison Tool						[159]
INFORMAS Food Provision Module						[163]
INFORMAS Food Composition Module						[164]
Children’s Menu Assessment						[165]
INFORMAS Food Labelling Module						[166]
INFORMAS Food Promotion Module						[167]
Checklist of Health Promotion Environments at Worksites (CHEW)						[168]
Store Layout and Marketing Analysis						[159]
Grocery store survey						[113,167]
ProDesirability Tool						[53]
American Time Use Survey (ATUS)						[169,170,171]
Photovoice						[172,173]

Color Coding: 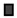 Objective measure; 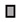 Perceived measure; 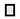 Both objective and perceived; ^†^ Measurement of promotion and quality only included in NEMS-R; * Measurement of convenience relates only to access for people with disabilities; INFORMAS: International Network for Food and Obesity/Non-communicable Diseases (NCDs) Research, Monitoring and Action Support.

## Appendix A

This scoring tool is adapted from Ahmed et al. (2019) [36]. Two coders are to apply the sustainability framework tool to score the food environment on the basis of observation. For each attribute, the coder is to assign a 0 for the absence of the attribute in the food environment and a 1 to indicate the presence of the attribute in the food environment.

**Table A1:** Availability of Foods with Sustainability Properties in the Food Environment.

Sustainability Dimension	Sustainability Attribute of Foods and Beverages Found in the Food Environment
**Ecological Dimension**	**Production quality:** The food environment contains food that supports production systems that cultivate for nutritional quality (crop quality). **Biodiversity, agrobiodiversity, and ecosystem services:** The food environment contains food that supports conservation and maintenance of biodiversity and agrobiodiversity as well as associated ecosystem services. **Sustainable agriculture:** The food environment contains food that supports sustainable agricultural practices and sustainable intensification that limit pesticide, herbicide and fertilizer use. **Local and seasonal foods:** The food environment contains food that are in season and are local. **Clean energy:** The food environment contains food produced through the use of clean energy and green or sustainable technologies **Soil, land, and water conservation and protection:** The food environment contains food produced and/or procured in ways that prevent contamination of soil, land, and water resources such as protecting watersheds from pollutants. **Low GHGE and climate resilience:** The food environment contains food produced and/or procured using methods with relatively low GHGE; cultivated in agricultural systems that manage for climate change/climate resilience.
**Economic Dimension**	**Distribution, supply chains, and transport:** The food environment contains food that supports direct sales between producers and consumers. **Food loss and waste:** The food environment minimizes loss of food waste across the food system from farm through fork. **Food packaging:** The food environment contains food that has minimum food packaging and/or encourages recycling. **Food system livelihoods:** The food environment contains food of which the production promotes livelihoods to support stakeholders in the food system from on farm and throughout food value chains. **Farmers’ markets and local food systems:** The food environment includes farmers’ markets, community supported agriculture (CSA), food cooperatives, and food hubs. **Food storage and preparation:** The food environment contains food of which the production and preparation avoids resource-intensive food storage of cold chain items and high-energy preparation such as the use of a microwave.
**Human Health Dimension**	**Food safety:** The food environment contains food that is safe and prevents foodborne illness, contamination, negative health influence of agriculture and diseases linked to chemicals and pesticide use. **Plant-based and nutrient-dense foods:** The food environment contains food that is plant-based and nutrient dense foods such as fruits, vegetables, and legumes.
**Socio-Cultural and Political Dimension**	**Equity issues:** The food environment contains food of which the production supports equity in the food system including on-farm, in market, trade, distribution, food service, and policy sectors. **Labor:** The food environment contains food that supports safe labor conditions and standards for workers in the food system. **Animal welfare:** The food environment contains food that supports healthy, comfortable, well nourished, and safe conditions for animals raised for livestock.

GHGE: Greenhouse gas emissions.

## Appendix B

The following survey questions ask individuals interacting with a specific food environment regarding their perceptions of the availability of foods with specific sustainability properties.

**Table A2:** Perceived Access to Foods with Sustainability Properties in the Food Environment.

Do You Think the Food Environment in Your Community Provides Adequate Access to the Following Types of Food?				
(1) Food that supports production systems that cultivate for nutritional quality (crop quality).				
☐ Yes, very high access	☐ Yes, good access	☐ Somewhat good access	☐ No, not available	☐ I don’t know
(2) Food that supports conservation and maintenance of biodiversity and agrobiodiversity as well as associated ecosystem services.				
☐ Yes, very high access	☐ Yes, good access	☐ Somewhat good access	☐ No, not available	☐ I don’t know
(3) Food that supports sustainable agricultural practices and sustainable intensification that limit pesticide, herbicide and fertilizer use.				
☐ Yes, very high access	☐ Yes, good access	☐ Somewhat good access	☐ No, not available	☐ I don’t know
(4) Food that is in season and are local.				
☐ Yes, very high access	☐ Yes, good access	☐ Somewhat good access	☐ No, not available	☐ I don’t know
(5) Food produced through the use of clean energy and green or sustainable technologies.				
☐ Yes, very high access	☐ Yes, good access	☐ Somewhat good access	☐ No, not available	☐ I don’t know
(6) Food produced and/or procured in ways that prevent contamination of soil, land, and water resources such as protecting watersheds from pollutants.				
☐ Yes, very high access	☐ Yes, good access	☐ Somewhat good access	☐ No, not available	☐ I don’t know
(7) Food produced and/or procured using methods with relatively low GHGE or cultivated in agricultural systems that manage for climate change/climate resilience.				
☐ Yes, very high access	☐ Yes, good access	☐ Somewhat good access	☐ No, not available	☐ I don’t know
(8) Food that has minimum food packaging and/or encourages recycling.				
☐ Yes, very high access	☐ Yes, good access	☐ Somewhat good access	☐ No, not available	☐ I don’t know
(9) Food that supports safe labor conditions and standards for workers in the food system.				
☐ Yes, very high access	☐ Yes, good access	☐ Somewhat good access	☐ No, not available	☐ I don’t know
(10) Food that supports equity in the food system including on-farm, in market, trade, distribution, food service, and policy sectors.				
☐ Yes, very high access	☐ Yes, good access	☐ Somewhat good access	☐ No, not available	☐ I don’t know

GHGE: Greenhouse gas emissions.
